# The discovery and identification of a candidate proteomic biomarker of active tuberculosis

**DOI:** 10.1186/1471-2334-13-506

**Published:** 2013-10-29

**Authors:** Jiyan Liu, Tingting Jiang, Liliang Wei, Xiuyun Yang, Chong Wang, Xing Zhang, Dandan Xu, Zhongliang Chen, Fuquan Yang, Ji-Cheng Li

**Affiliations:** 1 Institute of Cell Biology, Zhejiang University School of Medicine, 388, Yuhangtang Road, Hangzhou 310058, P.R. China; 2 The Sixth Hospital of Shaoxing, Shaoxing 312000, P.R. China; 3 Department of Respiratory Medicine, Tongde Hospital of Zhejiang, Zhejiang, China; 4 Laboratory of Proteomics, Institute of Biophysics, Chinese Academy of Sciences, Beijing 100101, P.R. China

**Keywords:** Tuberculosis, Biomarker, Proteomics, Mass spectrometry

## Abstract

**Background:**

Noninvasive and convenient biomarkers for early diagnosis of tuberculosis (TB) remain an urgent need. The aim of this study was to discover and identify potential biomarkers specific for TB.

**Methods:**

The surface-enhanced laser desorption ionization time of flight mass spectrometry (SELDI-TOF MS) combined with weak cation exchange (WCX) magnetic beads was used to screen serum samples from 180 cases of TB and 211 control subjects. A classification model was established by Biomarker Pattern Software (BPS). Candidate protein biomarkers were purified by reverse phase-high performance liquid chromatography (RP-HPLC), identified by MALDI-TOF MS, LC-MS/MS and validated using enzyme-linked immunosorbent assay (ELISA).

**Results:**

A total of 35 discriminating m/z peaks were detected that were related to TB (*P* < 0.01). The model of biomarkers based on the four biomarkers (2554.6, 4824.4, 5325.7, and 8606.8 Da) was established which could distinguish TB from controls with the sensitivity of 83.3% and the specificity of 84.2%. The candidate biomarker with m/z of 2554.6 Da was found to be up-regulated in TB patients, and was identified as a fragment of fibrinogen, alpha polypeptide isoform alpha-E preproprotein. Analysis in 22 patients with TB showed increased fibrinogen degradation product (FDP) (5,005 ± 1,297 vs. 4,010 ± 1,181 ng/mL, *P* < 0.05) and in 142 patients showed elevated plasma fibrinogen levels.

**Conclusions:**

A diagnostic model for TB with high sensitivity and specificity was developed using mass spectrometry combined with magnetic beads. Fibrinogen was identified as a potential biomarker for TB and showed diagnostic values in clinical application.

## Background

Tuberculosis (TB) is still a major infectious disease, threatening public health worldwide. Especially in developing countries, the epidemic situation of TB is alleviating slowly. The World Health Organization (WHO) has estimated that in 2011, there were 12 million prevalent cases and 8.7 million incident cases of TB in the world, and 1.4 million people died from TB. China is the second highest TB burden country with 1.2–1.6 million prevalent cases recorded in 2011 [1].

Early diagnosis is important for controlling TB [2]. Biomarkers play an irreplaceable role in early diagnosis, disease surveillance, treatment efficacy and prognostic evaluation of the disease. The detection of biomarkers is also a convenient, sensitive, specific, non-invasive, reproducible and inexpensive method [3]. At present, there are few effective biomarkers for early diagnosis of TB [4]. Therefore, the use of new technology to discover and verify more sensitive and specific biomarkers for early diagnosis of TB is a major challenge and urgent task for the disease control.

Detecting biomarkers in serum is an effective auxiliary means of diagnosis for disease [5]. The invasion of *Mycobacterium tuberculosis* (MTB) in the human body can change the expression of TB-associated proteins and release these proteins into the bloodstream through different pathways. Detection of serum antibodies in TB patients is precisely based on this principle [6]. However, as the TB antigens are varied and complex, the antibodies in TB patients may show a great variety. As a biomarker, not a single antigen can be recognized in the serum of all or most TB patients, and therefore a high sensitivity and specificity cannot be achieved [7]. The emergence of proteomics technology makes the analysis of all the proteins in the serum possible. Surface-enhanced laser desorption ionization time of flight mass spectrometry (SELDI-TOF MS), as a powerful proteomics technology integrating the technologies of chips/magnetic beads and mass spectrometry, can be directly used to detect crude body fluid samples without any labeling. This technology is simple, fast with high-throughput and high sensitivity [8,9]. Many protein biomarkers of certain diseases have been indicated by using SELDI-TOF MS to analyze the serum proteome [10-13].

The goal of this study was to screen for potential protein biomarkers in serum for the early diagnosis of TB using proteomics technology.

## Methods

### Patients and controls

We collected 391 serum samples from 180 patients with active pulmonary TB and 211 controls (91 healthy volunteers, 40 cases of lung cancer, 40 cases of pneumonia, and 40 cases of chronic obstructive pulmonary disease (COPD) from two separate sites: the Sixth Hospital of Shaoxing (Shaoxing, China) and Hangzhou Red Cross Hospital (Hangzhou, China). All TB patients were diagnosed according to combined clinical criteria from the WHO [14], including clinical, radiological, and bacteriological investigations and further confirmed by histopathological analysis. All the blood specimens were collected and preserved upon the first visit and before any treatment. The patients with hepatic, renal, metabolic and autoimmune disorders, endocrine, blood, nervous system diseases, malignant tumors, and long-term use of immunosuppressive agents were not included in the experiment. Both patients and controls were from the same ethnic (Han) population and lived in the same region (Southeast of China).

This study was approved by the Ethics Committees of the Faculty of Medicine (Zhejiang University, China), and informed consent was obtained from all subjects before collection of blood. The peripheral blood samples were collected from the TB patients and the controls in early morning without anticoagulation. Then the blood samples were allowed to clot for 1–2 hours prior to 4,000 g centrifugation for 10 minutes at 4°C to separate the serum out. The serum samples were aliquot and stored at -80°C for future analysis.

### SELDI-TOF MS analysis combined with WCX magnetic beads

In the sample pretreatment and proteomic analysis process, the serum samples from the diseased and control groups were randomized, and the investigator was blinded to their clinical manifestations. Serum samples were pretreated with WCX magnetic beads (Beijing SED Science & Technology, China). Briefly, 50 μL WCX magnetic beads were pre-activated with 100 μL binding buffer (50 mmol/L sodium acetate, pH 4.0) at 4°C in a magnet separator. Each serum sample was first diluted 1:2 with U9 solution (9 mol/L urea, 2% CHAPS [3-([3-cholamidopropyl] dimethylammonio)-1-propanesulfonate]), and incubated for 30 minutes at 4°C. Denatured serum samples were further diluted 1:40 in binding buffer. Then, 100 μL of the diluted serum sample was added to the activated magnetic beads, mixed, and incubated for 1 hour at 4°C, after which the beads were washed twice with 100 μL of binding buffer to remove non-selectively bound proteins. Following binding and washing, the bound proteins were eluted from the magnetic beads using 10 μL of 0.5% trifluoroacetic acid. Then, 5 μL of the eluted sample was diluted 1:2-fold in 5 μL of SPA (saturated solution of Sinapinic acid (SA) in 50% acetonitrile with 0.5% trifluoroacetic acid). Next, 2 μL of the resulting mixture was aspirated and spotted onto an 8-spot pre-structured sample Au-chip.

After air drying, protein crystals on the chip were scanned with the ProteinChip reader (model PBS IIc) (Ciphergen Biosystems, USA) to determine the masses and intensities of all peaks. The reader was set up as follows: mass range was set from 1,000 to 50,000 Da, optimized mass range was set from 1,000 to 15,000 Da, laser intensity was set at 265 and laser sensitivity was set at 7. The “All-in-one protein standard II” (Bio-Rad, USA) was used to obtain protein standard spectra for mass accuracy calibration.

### Detection and statistical data analysis

The profiling spectra of serum samples from the training set were normalized using total ion current normalization by Ciphergen ProteinChip Software (version 3.1). Peak labeling was performed by Biomarker Wizard software, version 3.1 (Ciphergen Biosystems). A two-sample t-test was used to compare mean normalized intensities between the case and control groups. Proteins with low *P*-values were selected, and the intensities of the selected peaks were transferred to Biomarker Pattern Software (BPS, Ciphergen Biosystems) to construct the classification tree of TB. Briefly, the intensities of the selected peaks were submitted to BPS as a “Root node”. Based on peak intensity, a threshold was determined by BPS to classify the root node into two child nodes. A sample would be labeled as “left-side child node” if the peak intensity of a blind sample was lower than or equal to the threshold. Meanwhile, if the peak intensities higher than the threshold, it would be marked as “right-side child node”. After rounds of decision-making, the training set was found to be discriminatory with the least error. All protein peak intensities of samples in the test set were evaluated by BPS using the classification model. The TB and control samples were then discriminated based on their proteomic profile characteristics. The sensitivity was defined as the probability of predicting TB cases, and the specificity was defined as the probability of predicting control samples. Accuracy was defined as the proportion of correct state classifications.

### Serum fractionation and purification of candidate peptide markers using RP-HPLC

Serum samples from the TB patients and the controls were selected for the purification of the candidate protein biomarkers. 100 μL serum samples were mixed with 600 μL acetonitrile (ACN) and 300 μL water for 30 minutes and centrifuged at 14,400 g for 30 minutes at 4°C. The supernatant fluid was collected and lyophilized dried to obtain 20 μL volume solution for further purification using RP-HPLC.

HPLC separation was performed using SCL-10AVP (Shimadzu, Japan) with a Ultimate® PAH C18 column (250 × 4.6 mm, 5 μm, Welch Materilas, Inc, MD, USA) and a C18 guard column (10 × 3 mm, Shimadzu, Japan). The mobile phase consisted of solvent A (water, 0.1% TFA) and solvent B (ACN, 0.09%TFA). The HPLC separation was achieved with a linear solvent gradient: 100% A (0 min)-20% B (10 min)-40% B (30 min)-70%B (70 min)- 100% B (75 min)-100% B(85 min) at a flow rate of 0.5 mL/min. The eluate emissions were detected at multiple wavelengths of 254, and 280 nm. Each peak fraction was collected and then analyzed using an AXIMA-CFRTM plus matrix-assisted laser desorption/ionization time-of-flight mass spectrometry (MALDI-TOF MS) (Kratos Analytical Co, UK) in linear mode to trace the candidate protein biomarkers with SA as the matrix. Then the target peptide (2554.6) containing fraction was obtained and used for the analysis of LC-MS/MS.

### Identification of candidate peptide biomarkers by LC-MS/MS

Target peptide (2554.6) containing fraction obtained above were loaded onto a home-made C18 column (100 mm × 100 μm) packed with Sunchron packing material (5 μm) and followed with nano-LC-ESI-MS/MS analysis without digestion. The LTQ mass spectrometer (Thermo Finnigan, USA) was operated in a data-dependent mode, in which the initial MS scan recorded the m/z ratios of ions over the mass range from 400–2000 Da firstly, and then the five most abundant ions were automatically selected for subsequent collision-activated dissociation.

The MS/MS data was searched using SEQUEST algorithm against the human protein database downloaded from the NCBI. The search was performed using a precursor mass tolerance of 3 Amu calculated using average isotopic masses and a fragment ion tolerance of 1.0 Amu. Variable modification was set for methionine with the addition of 15.999 Da to represent methionine oxidation. Enzyme cleavage specificity was set to no enzyme. The SEQUEST outputs were then analyzed using the commercial software ThermoFisher BioWorks (Rev.3.3.1sp2). The filter settings for peptides were as follows: XCorr: 1.9 (+1), 2.5(+2), 3.75(+3); Delta CN: >0.1; Sp: >500; Rsp ≤ 1.

### Measurement of serum fibrinogen degradation products (FDP) concentration and plasma fibrinogen concentration

The serum concentration of FDP was measured by enzyme-linked immunosorbent assay (ELISA). FDP in serum samples was quantified using a Human Fibrinogen Degradation Product ELISA kit (TSZ Scientific, USA) following manufacturer’s instructions. Test serum samples were diluted 1:200 in the dilution buffer supplied. The diluted Human FDP standards and test samples were added in duplicate to the wells of a microtiter plate coated with human FDP antibody.

The fibrinogen concentration in plasma was measured by the Clauss method using the STA®-Fibrinogen kit (Diagnostic Stago, France). Coagulation in STAGO compact automated analyzer (Diagnostic Stago, France) was determined to detect 142 cases of TB, which were recruited randomly from the First Hospital of Jiaxing (Jiaxing, China).

## Results

### Clinical evaluation of study subjects

The mean age and gender distribution were similar between the TB and non-TB control groups (*P* > 0.05). No differences were found in the number of individuals with BCG vaccination and HIV-negative between the two groups (*P* > 0.05) (Table 1). The detailed characteristics of the TB group are shown in Table 1. All TB patients showed different changes as inflammation, opacities, fibrosis and cavities in chest X-ray. Sputum culture for patients with TB was positive in 209 (65%) subjects with bacteriological analysis upon their first visit to hospital. For patients with negative sputum culture, the pathogens were identified in 49 (14.9%) patients with TB by brush biopsy, bronchoalveolar lavage, and biopsy of diseased pulmonary segment under fiberoptic bronchoscope. Typical histopathological changes of TB were identified in 7 (2.1%) patients with TB by percutaneous transthoracic needle biopsy. After the comprehensive diagnosis, such as combining clinical symptoms (fever, cough, expectoration, hemoptysis), radiological changes on chest X-rays, anti-tuberculosis antibody detection and molecular biological techniques to detect pathogen-specific sequence, 58 (18.0%) patients with TB received anti-tuberculosis treatment, and were confirmed based on treatment response. All TB patients involved in the study were followed up for one year to confirm the reliability of the results.

**Table 1:** Characteristics of TB patients and non-TB controls

	***Training set***	***Testing set***	***Clinical set***	***Total***
TB				
Total number of patients	120	60	142	322
Years range, age (media ± SD)	18-65(43.5 ± 12.0)	19-63 (44.4 ± 12.6)	20-62 (46.1 ± 10.5)	18-65 (44.7 ± 11.7)
Sex (female: male)	52:68	26:34	61:81	139:183
Abnormal chest radiograph	120 (100%)	60 (100%)	142 (100%)	322 (100%)
Sputum culture-positive	76 (63.3%)	41 (68.3%)	92 (64.8%)	209 (65.0%)
Sputum culture-negative	44 (36.7%)	19 (31.7%)	50 (35.2%)	113 (35.0%)
Pathogens identified	19 (15.8%)	8 (13.3%)	21 (14.8%)	48 (14.9%)
Histopathological changes of TB	3 (2.5%)	1 (1.7%)	3 (2.1%)	7 (2.1%)
Comprehensive dianoisis and confirmed by anti-tuberculosis treatment response	22 (18.3%)	10 (16.7%)	26 (18.3%)	58 (18.0%)
BCG vaccination	52 (43.3%)	27 (45.0%)	55 (39%)	134 (41.6%)
HIV-negative	120 (100%)	60 (100%)	142 (100%)	322 (100%)
Non-TB controls				
Total number of control subjects	120	91		211
Healthy volunteers	60 (50.0%)	31 (34.1%)		91 (43.1%)
Lung cancer	20 (16.7%)	20 (22.0%)		40 (19.0%)
Pneumonia	20 (16.7%)	20 (22.0%)		40 (19.0%)
Chronic obstructive pulmonary disease	20 (16.7%)	20 (22.0%)		40 (19.0%)
Years range, age (media ± SD)	20-65 (46.5 ± 13.9)	19-64 (46.1 ± 14.4)		19-65 (46.3 ± 14.1)
Sex (female: male)	48:72	38:53		86:125
BCG vaccination	53 (44.2%)	39 (42.9%)		92 (43.6%)
HIV-negative	120 (100%)	91 (100%)		211 (100%)
Total	240	151		533

*TB* tuberculosis, *BCG* Bacillus Calmette-Guérin, *HIV* human immunodeficiency virus.

The subjects in the TB and non-TB control groups were randomly divided into two sets: the training set and the blinded test set (Table 1). All characteristics between the two sets were tested for statistical significance to ensure that nothing outside the main differentiating factor confounded the results.

### Serum protein profiles and data processing

**Figure 1:**
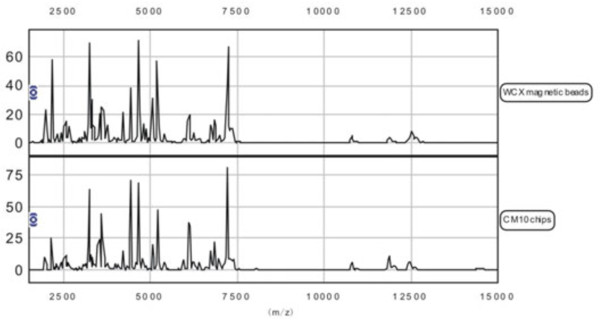
**Representative protein spectrum of tuberculosis sample detected by SELDI-TOF MS.** Protein spectrum of tuberculosis sample detected by SELDI-TOF MS showing the protein mass/charge between 1,500 and 15,000. SELDI-TOF MS: surface-enhanced laser desorption/ionization time-of-flight mass spectrometry; WCX magnetic beads: SELDI-TOF MS combined with WCX magnetic beads; CM10 chips: SELDI-TOF MS combined with CM10 chips; m/z: mass-to-charge ratio.

We analyzed the variance between all m/z peak intensities after calibration, smoothing, alignment, and normalization, and the coefficient of variation (CV) was found to be less than 10% for all the selected mass peaks (Additional file 1: Figure S1). Up to 251 protein peaks per spot were detected between m/z 1500 and m/z 15,000 and the protein peaks showed the effectiveness of the SELDI technology combined with WCX magnetic beads separation of low-molecular weight proteins (<15,000) (Figure 1).

The protein profile of the 240 serum samples from the training set (120 cases of TB, 20 cases of lung cancer, 20 cases of pneumonia, 20 cases of COPD, and 60 healthy volunteers) were analyzed with Biomarker Wizard software, and 35 m/z peaks were found to discriminate between patients with TB and non-TB control subjects (*P* < 0.01, Fold ≥ 1.5) (Table 2). Among these peaks, 16 were down-regulated and 19 were up-regulated in patients with TB compared to the non-TB control subjects.

**Table 2:** The 35 discriminating m/z peaks between patients with TB and non-TB control subjects

***m/z***	***P***^**a**^	**Fold**	***m/z***	***P***^**a**^	**Fold**	***m/z***	***P***^**a**^	**Fold**
4824.4^b^	0	+6.8	1819.4	1.0 × 10^-6^	+1.5	5199.8	1.3 × 10^-4^	-2.4
6924.9	1.0 × 10^-10^	-1.7	1934.0	2.1 × 10^-6^	+1.6	6075.4	3.0 × 10^-4^	-1.5
2513.0	1.0 × 10^-10^	+1.6	16015.9	4.7 × 10^-6^	-1.5	5733.5	4.6 × 10^-4^	-1.7
1662.4	2.0 × 10^-10^	+1.5	1687.1	1.3 × 10^-5^	+2.7	1600.3	8.5 × 10^-4^	+7.5
2958.0	9.7 × 10^-9^	+1.8	7752.3	2.1 × 10^-5^	-2.0	2603.8	0.001	+2.3
4537.9	1.4 × 10^-8^	+1.6	1575.5	2.2 × 10^-5^	+1.6	5896.9	0.002	-1.8
6098.2	3.5 × 10^-8^	-1.6	1596.3	2.3 × 10^-5^	+1.5	2012.2	0.003	-2.4
2554.6^b^	4.1 × 10^-8^	+5.8	10263.8	4.2 × 10^-5^	+2.4	3242.4	0.003	-1.6
2310.2	9.3 × 10^-8^	+1.5	8606.8^b^	4.5 × 10^-5^	+1.9	1799.3	0.008	+1.6
3959.5	4.0 × 10^-7^	-1.9	5129.8	5.7 × 10^-5^	-2.1	2010.3	0.008	-4.3
15855.9	4.4 × 10^-7^	-1.6	1615.6	6.2 × 10^-5^	+1.7	2339.5	0.009	+2.9
9269.9	6.3 × 10^-7^	-1.9	5325.7^b^	1.0 × 10^-4^	-2.6	N/A	N/A	N/A

*m/z* means mass-to-charge ratio. ^a^ Generated by peak comparison between TB and non-TB controls. ^b^ Peak selected as biomarkers for TB diagnostic model. +fold change (up-regulated), - fold change (down-regulated).

*P* < 10^-10^ regarded as 0.

**Figure 2:**
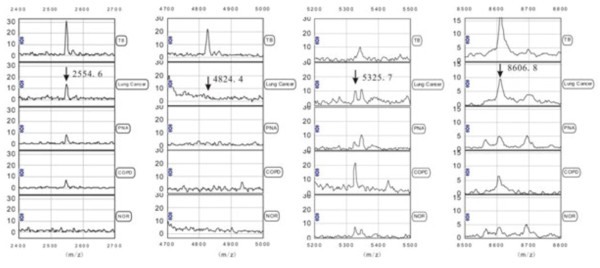
**Differential expression of SELDI-TOF MS peaks in serum samples.** Peaks with mass/charge of 2554.6, 4824.4, 5325.7, and 8606.8 were detected by SELDI-TOF MS in serum samples from patients with tuberculosis, control subjects with lung cancer, chronic obstructive pulmonary disease, pneumonia, and healthy control subjects. PNA: pneumonia; NOR: healthy control subjects; COPD: chronic obstructive pulmonary disease; SELDI-TOF MS: surface-enhanced laser desorption/ionization time-of-flight mass spectrometry; m/z: mass-to-charge ratio.

**Figure 3:**
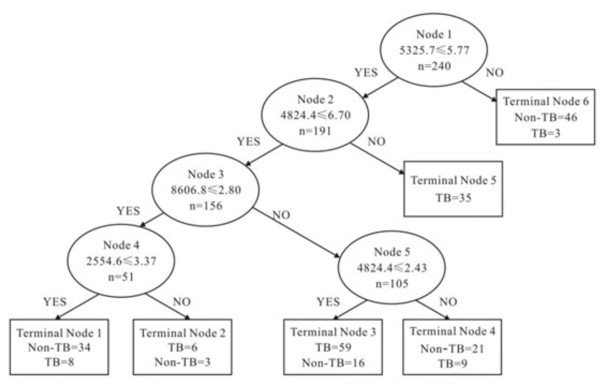
**Decision trees in the diagnostic model for tuberculosis.** Four peaks, mass/charge 2554.6, 4824.4, 5325.7 and 8606.8 were chosen to set up the decision tree by the Biomarker Patterns Software. The diagnostic model shows the tree structure and sample distribution of the training set.

According to the variable importance, the 2554.6, 4824.4, 5325.7, and 8606.8 m/z peaks were most important. The four peaks were selected by the BPS to construct a classification tree (Figure 2). Figure 3 shows the tree structure and sample distribution. The classification tree using the combination of the four peaks identified 120 patients with TB and 120 non-TB controls with a calculated sensitivity of 83.3% and a specificity of 84.2% (overall accuracy 83.8%). We used 151 samples of the blinded test set, including 60 from patients with TB, 20 cases of lung cancer, 20 cases of pneumonia, 20 cases of COPD and 31 cases of healthy volunteers to test the TB diagnostic model. The classification tree discriminated the TB samples from the control samples with an accuracy of 80.1% (75.0% sensitivity, 83.5% specificity) (Table 3).

**Table 3:** Prediction results of the diagnostic model for TB

***Group***	***Samples***	***Cases***	***Correct-classed***	***Diagnosis rate%***
Training set	TB	120	100	83.3%
Testing set	Non-TB	120	101	84.2%
	TB	60	45	75.0%
	Non-TB	91	76	83.5%

### Identification of a fragment (2554.6) of fibrinogen as a potential response marker for TB

**Figure 4:**
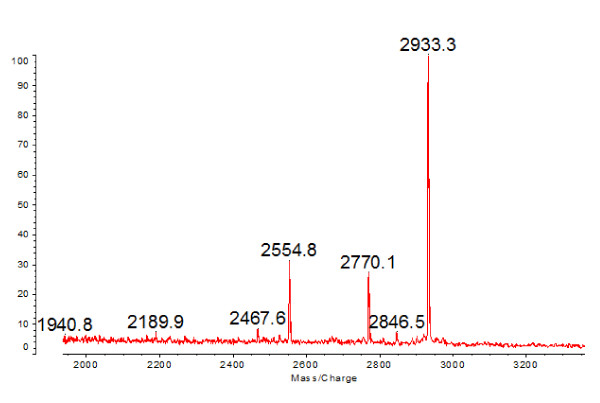
**MALDI-TOF MS spectra of the potential peptide biomarker containing fraction.** Serum samples from TB patients were used for the purification of the up-regulated peptide biomarker (mass/charge 2554.6) using HPLC. The purified fraction contains the target peptide (2554.6) and four other peptides peaks (2467.5, 2770.1, 2846.5 and 2933.3).

**Figure 5:**
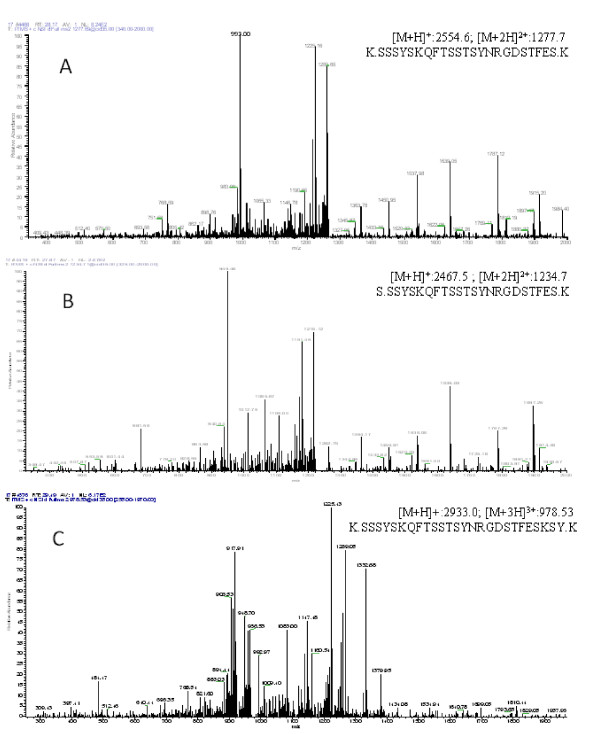
**Results of the identification of target peptide (2554.8) containing fraction by LC-MS/MS.** The three major peptides were identified as fragments of the same protein fibrinogen. **(A)** MS/MS spectrum of peptide (K.SSSYSKQFTSSTSYNRGDSTFES.K, [M + H]^+^: 2554.6; M + 2H]^2+^:1277.7); **(B)** MS/MS spectrum of peptide (S.SSYSKQFTSSTSYNRGDSTFES.K, [M + H]^+^: 2467.5; [M + 2H]^2+^:1234.7); **(C)** MS/MS spectrum of peptide (K.SSSYSKQFTSSTSYNRGDSTFESKSY.K, [M + H]^+^:2933.0; [M + 3H]^3+^: 978.53).

Serum samples were used for the purification of the candidate protein biomarker using ACN and RP-HPLC. The result of MALDI-TOF MS analysis showed one target peptide (2554.6) containing peak fraction also contained four other peptides (2467.5, 2770.1, 2846.5 and 2933.3) (Figure 4) even they were not detected by SELDI-TOF-MS. Then the peptide mixture was analyzed by nano-LC-MS/MS (Figure 5) and the three major peptides were identified as fragments of protein fibrinogen, alpha polypeptide isoform alpha-E preproprotein [Homo sapiens] (gi|4503689|ref|NP_000499.1| [MASS = 94973]) (Table 4). Some other small peptides were also identified as fragments of fibrinogen from the same fraction (not shown in Table 4).

**Table 4:** The identified peptides from fibrinogen

**Peptides identified from fibrinogen**	**MH+**	**z**	**XC Score**	**DeltaCn**
S.SSYSKQFTSSTSYNRGDSTFES.K	2467.50	2	3.18	0.30
**K.SSSYSKQFTSSTSYNRGDSTFES.K**	**2554.57**	**2**	**4.37**	**0.43**
K.SSSYSKQFTSSTSYNRGDSTFESKSY.K	2933.00	3	4.50	0.32

**Figure 6:**
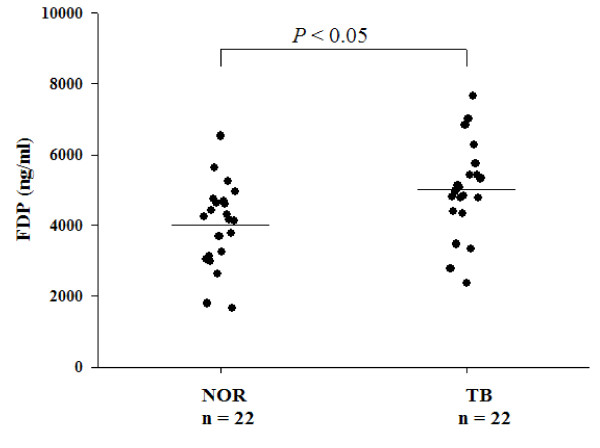
**Serum levels of FDP in healthy controls and TB patients measured by ELISA.** A *P*-value of less than 0.05 indicates statistical significance using the t-test. NOR: healthy control subjects. FDP: fibrinogen degradation product; TB: tuberculosis; ELISA: enzyme-linked immunosorbent assay.

To evaluate the FDP levels in serum, an ELISA was done on serum from 22 patients with TB, and 22 healthy volunteers, which were selected randomly from the testing set. The levels of FDP in the TB group was higher than that of the healthy group (5,005 ± 1,297 vs. 4,010 ± 1,181 ng/mL, *P* < 0.05) (Figure 6). To measure the plasma fibrinogen levels in TB patients, the Clauss method was used for 142 confirmed TB cases. The detailed characteristics are shown in Table 1. The results showed higher levels of plasma fibrinogen in patients with TB (5.45 ± 1.65 g/L), compared to the normal group (2.0-4.0 g/L).

## Discussion

Pathogenic mechanisms and the pathological changes caused by MTB invasion in human are all based on protein expression and protein-protein interactions. The host defense system is triggered mainly through the immune/inflammatory response [15]. TB infection can lead to the synthesis of TB-associated proteins which appear in the blood circulation through a variety of pathways such as direct secretion of proteins at the lesions affected by the MTB, stimulated production of reactive proteins, or production of proteins due to the disintegration of the MTB [16,17]. These TB-associated proteins can be used as potential diagnostic markers for discriminating TB patients.

In biomarker research with SELDI-TOF MS, the aim is to identify peak intensities that are different between case and control samples, and the reproducibility of peak intensities is of highest importance. However, poor reproducibility has been considered one of the problems with SELDI-TOF MS. In our study, strict Standard Operating Procedures, internal and external control were combined for data quality and reproducibility. In internal control method, one point was randomly selected for each Au chip to perform the same experiment with quality control serum. The “All-in-one protein standard II” was used as the external control to obtain protein standard spectra for mass accuracy calibration. All TB and control samples were detected by SELDI-TOF MS using the same batch of magnetic beads, the same Au-chip and on the same equipment. The same procedures were followed within one week to ensure experimental repeatability and reliability.

A total of 35 discriminating m/z peaks were detected that were related to TB (*P* < 0.01, Fold ≥ 1.5). The model based on the four biomarkers (2554.6, 4824.4, 5325.7, and 8606.8 Da) was established which could distinguish the TB patients from the controls. In the blinded test set, the results yield a sensitivity of 75.0% and a specificity of 83.5%.

Compared to previous similar studies [16,18,19], the candidate markers we found were not the same. One of the possible reasons may be the use of a different type of magnetic beads in our detection method. Previous studies have adopted the CM10 (weak cation exchange) protein chip for the application of SELDI technology in TB biomarkers discovery. In the present study, we applied the WCX magnetic beads. Although both are weakly cationic, as a new protein separation technology, the WCX magnetic beads provide a great flexibility for fractionation of complex biological samples. It has been successfully used in the separation and purification of various proteins in the body fluids such as serum, plasma and amniotic fluid [20-22]. During the pilot study, we compared the SELDI spectra of the serum in TB patients by WCX magnetic beads and CM10 protein chips. The results showed that the WCX magnetic beads could screen more proteins from serum samples of TB patients, with higher accuracy of the protein peaks and had stronger ability of protein capturing (Figure 1). Therefore, WCX magnetic beads are more useful to discover new protein biomarkers in serum.

Another reason may be the difference in racial genetic factors. In a study reported by Agranoff *et al*. for TB patients [18], most cases in the TB group were African patients, while the majority of the control groups were Caucasian, which might result in bias. In this study, we attempted to find new TB diagnostic markers in the Chinese Han population.

Meanwhile, the candidate protein markers were further identified in our study. During the pilot study we tried the adsorption method enriched using WCX magnetic beads and ACN precipitation method. Finally, the ACN precipitation method with ACN: H_2_O: serum ratio of 6:3:1 was used in serum sample precipitation, which could enrich the majority of low-molecular-weight proteins or peptides in the supernatant. By tracking with MALDI-TOF MS, the target peptides were purified and then analyzed by nano-LC-MS/MS. One candidate peptide peak (2554.6 Da) and other two peptides (2467.6, 2933.3 Da) were identified as the fragments of fibrinogen, alpha polypeptide isoform alpha-E preproprotein [Homo sapiens] (gi|4503689|ref|NP_000499.1| [MASS = 94973]), which indicated that the levels of FDP in the TB patients group may be higher than that of the non-TB group. In our study, the results showed a higher level of FDP in TB patients (5,005 ± 1,297 vs. 4,010 ± 1,181 ng/mL, *P* < 0.05) than that of healthy volunteers. Robson *et al.*[23], in a study of the hemostatic profiles of patients with acute TB, reported an increased FDP levels. The experimental results were consistent with ours. Increased FDP in the blood of TB patients indicated that the fibrinolytic system was activated.

One of the possible reasons of fibrinolytic system activation is the increase of fibrinogen levels in the blood. The activated fibrinogenase in the fibrinolytic system enters into the blood in large quantity, which will cause the increase of FDP in serum. This is part of the body’s defense function [24,25]. Robson *et al.*[23] reported an increase in FDP levels, concurrent with elevated levels of fibrinogen. Fibrinogen is an acute-phase reactant and its production rate may increase greatly as a result of various essentially non-specific stimuli [26-28]. Turken *et al*. [29], in a study of the hemostatic changes in active pulmonary TB, found that elevated plasma fibrinogen levels appear to induce a hypercoagulable state. Others also reported significantly higher levels of plasma fibrinogen in Nigeria pulmonary TB patients [30]. Similarly, in our study, analysis in 142 patients with TB showed increased plasma fibrinogen levels (5.45 ± 1.65 g/L). It has been postulated that the vascular endothelium could be primed as a result of interaction between mycobacterial products and the host monocyte-macrophage system, which then synthesis large amounts of cytokines, such as tumor necrosis factor-alpha and IL-6. These cytokines induce hepatic acute-phase responses that alter the levels of coagulation proteins such as fibrinogen [29,31,32].

In addition, it has been reported that plasminogen (Plg)- a member of the fibrinolytic system, can be bound to and immobilized on the microbes’ surface by Plg receptors and activated by host or pathogen activators to generate the proteolytic enzyme plasmin (Plm), which turns the microbes into proteolytic organisms, thereby augmenting their invasive potential. Bacteria can also exploit the molecules of the fibrinolytic system to avoid the innate immune response, and the fibrinolytic system itself can modulate the inflammatory response induced by the pathogen [33,34]. MTB has been demonstrated to have high number of Plg receptors, which can be activated to Plm by host activators, suggesting that this interaction could have a role in host-bacteria relationship [34,35]. Therefore, we speculate that activation of the fibrinolytic system is closely associated with the onset and progress of TB.

In our study, the blinded test of the diagnostic model for TB yielded a sensitivity of 75.0% and a specificity of 83.5%. The results showed that 25% of TB patients were not sensitive to the diagnostic model, and 16.5% of the cases were false negative. In addition to the technical methods and the statistical discrepancy, the lack of a gold standard diagnosis in 100% of the cases is clearly a limitation of our study. Only 65% of TB cases were culture positive proven cases, and a minority of culture negative cases may not have TB. Although the patients with negative sputum culture were diagnosed based on comprehensive combined clinical and radiological findings, and TB treatment outcomes, but there may be other pulmonary infectious diseases which may mimic pulmonary TB.

Meanwhile, the up-regulated peak at 2554.6 m/z was identified as a fragment of fibrinogen in our study. A significant minority (35%) of the cases in the dataset used to identify the biomarker were not defined by the diagnostic gold standard. So in large sample clinical studies, sputum culture is the gold standard for diagnosis of TB.

## Conclusions

The model we constructed using the protein peaks at 2554.6, 4824.4, 5325.7, 8606.8 m/z could successfully distinguish the TB patients from the controls. The peak at 2554.6 m/z was identified as a fragment from fibrinogen. In addition, we found increased levels of fibrinogen and FDP in TB patients, reflecting the activation of fibrinolytic system from another perspective. Although further experiments and larger studies are indispensable to prove the reliability of the proteins identified in this study, our results will help in the diagnostic evaluation and response to therapy in patients with TB.

## Competing interests

The authors declare that they have no competing interests.

## Authors’ contributions

JCL conceived the study and designed the experiments. JL,TJ, LW, XY and XZ collected the serum samples. JL, T J, CW, ZC and DX analyzed the data with suggestions by JCL. JL and FY finished the mass spectrometry analysis. JL and JCL wrote the manuscript. All authors read and approved the final manuscript.

## Pre-publication history

The pre-publication history for this paper can be accessed here:

http://www.biomedcentral.com/1471-2334/13/506/prepub

## Supplementary Material

Additional file 1: Figure S1 Supplementary file providing additional Figures S1 in one PDF file. Click here for file
