# Supplementary material for: The discovery and identification of a candidate proteomic biomarker of active tuberculosis
Source: BMC Infect Dis. 2013 Oct 29;13:506. doi: 10.1186/1471-2334-13-506 (PMC3870977; doi:10.1186/1471-2334-13-506)

Figure S1. A 2-spot test showed good reproducibility. The coefficient of variation (CV) of all selected mass peaks was <10%.

Reproducibility was evaluated with four mixed serum samples from the healthy control subjects of blood type O (two women and two men). The mixed serum samples were spotted on two spots. We analyzed the variance between all m/z peak intensities after calibration, smoothing, alignment, and normalization, and the coefficient of variation (CV) was found to be <10% for all selected mass peaks.

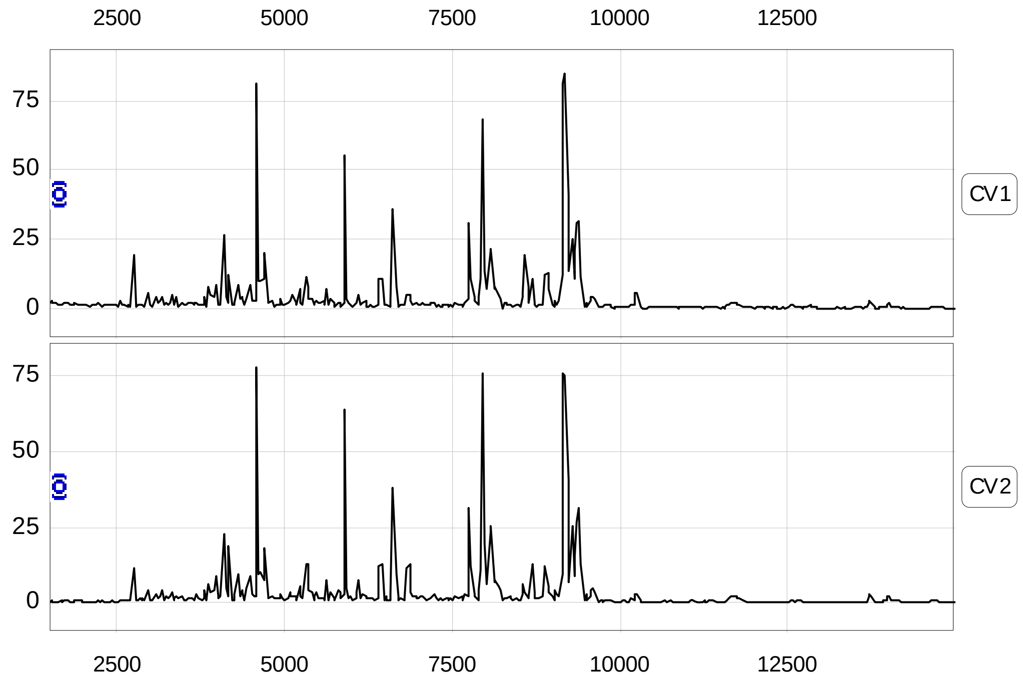

Supplement: Additional file 1: Figure S1 — Supplementary file providing additional Figures S1 in one PDF file. [file 1471-2334-13-506-S1.pdf]
